# Neural and Synaptic Defects in *slytherin*, a Zebrafish Model for Human Congenital Disorders of Glycosylation

**DOI:** 10.1371/journal.pone.0013743

**Published:** 2010-10-29

**Authors:** Yuanquan Song, Jason R. Willer, Paul C. Scherer, Jessica A. Panzer, Amy Kugath, Emmanuel Skordalakes, Ronald G. Gregg, Gregory B. Willer, Rita J. Balice-Gordon

**Affiliations:** 1 Department of Neuroscience, University of Pennsylvania School of Medicine, Philadelphia, Pennsylvania, United States of America; 2 Wistar Institute, Philadelphia, Pennsylvania, United States of America; 3 Department of Biochemistry and Molecular Biology, University of Louisville, Louisville, Kentucky, United States of America; National Institutes of Health (NIH), United States of America

## Abstract

Congenital disorder of glycosylation type IIc (CDG IIc) is characterized by mental retardation, slowed growth and severe immunodeficiency, attributed to the lack of fucosylated glycoproteins. While impaired Notch signaling has been implicated in some aspects of CDG IIc pathogenesis, the molecular and cellular mechanisms remain poorly understood. We have identified a zebrafish mutant *slytherin* (*srn*), which harbors a missense point mutation in GDP-mannose 4,6 dehydratase (GMDS), the rate-limiting enzyme in protein fucosylation, including that of Notch. Here we report that some of the mechanisms underlying the neural phenotypes in *srn* and in CGD IIc are Notch-dependent, while others are Notch-independent. We show, for the first time in a vertebrate *in vivo*, that defects in protein fucosylation leads to defects in neuronal differentiation, maintenance, axon branching, and synapse formation. *Srn* is thus a useful and important vertebrate model for human CDG IIc that has provided new insights into the neural phenotypes that are hallmarks of the human disorder and has also highlighted the role of protein fucosylation in neural development.

## Introduction

Congenital disorder of glycosylation, type IIc (CDG IIc), also known as leukocyte adhesion deficiency II (LAD II) or Rambam-Hasharon syndrome (RHS), is an autosomal recessive syndrome, characterized by recurrent infections, persistent leukocytosis, severe mental retardation and slowed growth [1], [2]. The immunodeficiency that is a hallmark of these syndromes is believed to be caused by dysregulated fucose metabolism, resulting in the absence of all fucosylated glycans on the cell surface [1], [2]. The gene responsible for CDG IIc has been identified as GDP-fucose transporter (*FUCT1*) [3], [4], which translocates GDP-fucose from the cytosol into the Golgi lumen for fucosyltransferase-catalyzed reactions during the modification of glycans.

Several animal models have been generated to study the pathogenesis of CDG IIc: *FX* locus null mice, lacking an enzyme in the *de novo* GDP-fucose synthesis pathway [5], *Gfr* (homologous to *FUCT1*) null flies [6] and *Fuct1* null mice [7]. *Gfr* null flies display *Notch*-like phenotypes during wing development and reduced Notch fucosylation, suggesting that Notch deficiency may be responsible for some of the developmental defects in CDG IIc patients [6]. However, despite the neurodevelopmental and cognitive dysfunction prominent in CDG IIc patients, the anatomical, cellular and molecular abnormalities within the nervous system have not been well documented, and the mechanisms underlying this and other neural phenotypes remain unexplored.

A large body of literature has demonstrated an important role for Notch-Delta signaling in neuronal and glial specification, neuronal maturation and learning and memory [8]. Specifically, in zebrafish, Notch-Delta signaling has been shown to regulate neurogenesis and gliogenesis. For instance, deficiency of Notch1a as in *deadly seven* (*des*) mutants resulted in increased primary motor neuron and Mauthner neuron number [9]; deficiency of Delta A as in *dla* mutant caused excessive primary motor neurogenesis at the expense of secondary motor neurons, some ventral interneurons and oligodendrocytes [10], [11], [12]; mutation of *Mind Bomb* (an E3 ubiquitin ligase for Delta) as in *mib* resulted in a severe neurogenic phenotype together with the loss of oligodendrocytes [12], [13]. While some studies support the involvement of Notch signaling in the balance of excitatory/inhibitory synapses in hippocampus [14] and during synaptic plasticity [15], whether Notch-Delta signaling modulates synaptogenesis is unknown.

Here we report the genetic, cellular and molecular characterization of a zebrafish mutant *slytherin* (*srn*). Previously, we have identified *srn* as a synaptogenic mutant that exhibits abnormal swimming behavior, has increased primary motor neurons and aberrant neuromuscular synaptogenesis [16]. We have found that the *srn* mutation resides in GDP-mannose 4, 6-dehydratase (GMDS), the first and rate-limiting enzyme in the fucose metabolism pathway. Because dysfunction of the same pathway is responsible for human CDG IIc, we performed cellular and molecular analyses that suggest that *srn* has Notch-Delta dependent and independent defects, consistent with a general defect in protein fucosylation that affects several aspects of neural development.

## Materials and Methods

### Zebrafish maintenance and mutants

Zebrafish were raised and maintained under standard conditions. The *srn* allele was previously described [16]. The *des^b420^* allele was obtained from Dr. Christine Beattie, *Tg(hsp70l:GAL4)* and *Tg(UAS:myc-notch1a-intra)* [17] from Dr. Bruce Appel, and *dla^hi781^* and *mib^hi904^* alleles from Zebrafish International Resource Center, University of Oregon.

### Positional cloning of srn

Genetic mapping of mutant loci was performed as described [18]. New simple sequence repeat (SSR) markers DKEY-25E12-SSR2 (forward, 5′-gcacacatgcatacgttcag-3′; reverse, 5′-tcccaaagtgaaagggtgag-3′) and DKEY-177P2-SSR4 (forward, 5′-cctgagggtcaggagagtaatg-3′; reverse, 5′-gaactaacactttcacaaacaccaa-3′) were used to define the interval that contained the mutation. PCR products containing the entire ORF of *gmds* (accession # NM_200489) were generated with the primers 5′-cggatgtgtttgcatccgta-3′ and 5′-tcacatgaattaaacggcat-3′ for both mutant and WT (WT) cDNAs, cloned into pCR 4-TOPO (Invitrogen), and sequenced for validation.

### RNA extraction and quantitative RT-PCR (qRT-PCR)

RNA was extracted (20 embryos) with the RNeasy kit (Qiagen, Inc.). *hes5* was amplified with primers 5′-gaaagccagtggtggaaaag-3′ and 5′-gaaagccagtggtggaaaag-3′. *her4* was amplified with primers 5′-cctggagatgacgcttgatt-3′ and 5′-cactgggcactgagacagaa-3′. *heyl* was amplified with primers 5′- gcgatacctcagctctttgg-3′ and 5′-ggagaggatccagctcactg-3′. *β-actin1* was amplified with primers 5′-tgaatcccaaagccaacagagaga-3′ and 5′-tcacgaccagctagatccagacg-3′. qRT-PCR was performed with the SuperScript® III Platinum® SYBR® Green One-Step qPCR Kit w/ROX (Invitrogen) and data was analyzed with 7500 Real-Time PCR System software (Applied Biosystems) using the 2^-ΔCT^ method.

### Whole mount in situ hybridization

*gmds* cDNA was cloned into pBluescript (Stratagene). The plasmid was linearized and anti-sense and sense probes were made with the Dig RNA labeling kit SP/T7 (Roche). *hes5 in situ* probe was generated with primers 5′-tggctcctgcgtatatgactgaat-3′ and 5′-gcggctcctgcttgatgtgt-3′. *her4 in situ* probe was generated with primers 5′-tctgatcctgacggagaactg-3′and 5′-ttcagtccatgccaatctca-3′ [19]. *heyl in situ* probe was generated with primers 5′-tcaaccacagcctgtcagag-3′ and 5′-caggggaatgctgttgaagt-3′ [20]. *In situ* hybridization was performed as described previously [16].

### GDP-fucose rescue and gmds mRNA and morpholino injection

GDP-fucose (50 mM in water (pH = 7) with 0.1% phenol red as a tracer) was injected directly into 1–2 cell stage embryos collected from crosses of *srn* carriers. *Gmds-gfp* mRNAs (WT and *srn*) were injected into embryos from WT and *srn* incrosses at the 1–2 cell stage at ∼200 pg. The morpholino antisense oligonucleotide (Gene Tools) targeting the *gmds* exon5-intron5 junction (CGTATGTTTGCTGACCATAAGGCGA) was injected at the 1–2 cell stage at ∼4 ng.

### Expression of Notch1a by heat-shock induction and rescue of gmds morphant phenotypes

To induce expression of constitutively active Notch1a (Notch1a intracellular domain, NICD), embryos were collected from matings of heterozygous *Tg(hsp70l:GAL4)* and *Tg(UAS:myc-notch1a-intra)* adults and raised at 28.5°C. At 11 hpf, embryos were heat-shocked at 39°C for 30 minutes and then returned to 28.5°C until the desired stage of development [21]. To determine whether NICD rescues *srn* phenotypes, *gmds* MO was injected into NICD transgenic embryos and the phenotypes were compared to NICD transgenic embryos alone, WT, *srn* and *gmds* MO embryos.

### DAPT treatment

Embryos were dechorionated with forceps at 6 hpf and placed in DAPT (N-[N-(3,5-Difluorophenacetyl-l-alanyl]-S-phenylglycine-t-butyl ester; Calbiochem) solution at 28.5°C until the appropriate stage, as previously described [22]. For experiments, 50 µM (medium dose) and 100 µM (high dose) DAPT in embryo medium containing 1% DMSO was used. Control embryos were incubated in an equivalent concentration (1%) of DMSO.

### Immunostaining, AAL staining and labeling of retinotectal projections

Embryos were anesthetized, fixed and immunostained as described previously [16] using antibodies against SV2, Zn5, 3A10, Islet1/2, F59 (all from Developmental Studies Hybridoma Bank, Univ. of Iowa) and/or goldfish GFAP [23] (gift from Drs. S. Nona and J. Scholes, Univ. of Sussex, United Kingdom) and fluorescently conjugated secondary antibodies (Jackson Labs, Inc.). Fluorescently conjugated α-bungarotoxin (Molecular Probes, Inc.) was used to label AChRs [16]. TUNEL staining was performed according to the manufacturer's instructions (Chemicon, Inc.). Fucosylated proteins were visualized in 48 hpf embryos using a biotinylated fucose-specific lectin, *Aleuria Aurantia* lectin (AAL; 20 µg/ml; Vector Labs; [24], [25] followed by Alexa 488 conjugated strepavidin (20 µg/mL; Molecular Probes). The number of Zn5+ cells was counted at 20 µm intervals along the rostral-caudal axis of several spinal cord hemisegments and compared statistically using Kolmogorov-Smirnov test. Retinal ganglion cell axon projections to the optic tectum were labeled as described [26].

Unless otherwise stated, each immunostaining or dye labeled figure panel is a single plane projection of a confocal z-stack of 20–160 1 µm thick planes (Leica TCS 4D). Presynaptic vesicles, AChR clusters and the co-localization of these two markers were measured from using interactive software (Metamorph).

## Results

### External phenotype, genetic cloning and mRNA rescue of slytherin

**Figure 1:**
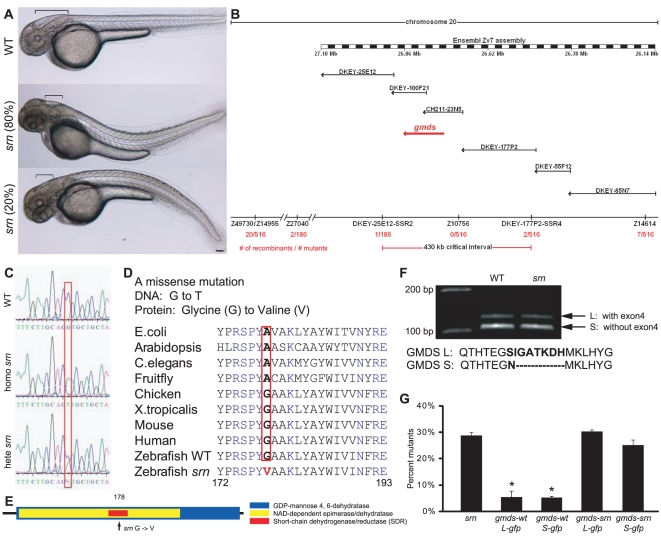
*Slytherin* external phenotype, genotype, cloning and mRNA rescue of *srn* mutants. **A.** External *srn* phenotypes at 48 hpf include a bent tail (80% dorsal (534 embryos, 8 carrier pairs)) and aberrant hindbrain formation (brackets). Scale bar  = 100 µm. **B.** Genetic and physical map of the *srn* locus (red arrow), including SSLP markers, number of recombinants, BAC clones and megabase positions from Ensembl Zv7. **C**, **D.** In *srn*, *Gmds* mutation is G to T (C, red box) resulting in a Glycine to Valine conversion (D, red box). GMDS amino acid sequence is highly conserved. **E.** Schematic of *srn* mutation in the short-chain dehydrogenase/reductase (SDR) domain of GMDS. **F.** Two splice variants exist in *gmds* mRNA, with (*gmds-L*, 377 aa) or without (*gmds-S*, 370 aa) exon 4. *Gmds* alternative splicing is not altered in *srn* mutants. **G.** Injection of *gmds* mRNA rescues *srn* mutants. Compared to uninjected embryos, 28.6±1.2% of embryos were mutant when scored by external phenotypes (3413 embryos, 27 carrier pairs). In embryos injected with WT *gmds-gfp* mRNA, the percentage of mutants scored by external phenotypes was significantly decreased, to ca. 5% (*gmds-wtL-gfp* 5.4±2.5%, 401 embryos, 3 carrier pairs; *gmds-wtS-gfp* 5.1±0.6%, 587 embryos, 4 carrier pairs; one-way ANOVA, followed by Dunn's pairwise comparison, p<0.05). The percentage of embryos with mutant external phenotypes was unchanged in embryos injected with mutant *gmds-gfp* mRNA (*gmds-srnL-gfp* 30.2±0.9%, 387 embryos, 3 carrier pairs; *gmds-wtS-gfp* 25.1±1.9%, 516 embryos, 4 carrier pairs). This mRNA rescue experiment confirms that *gmds* is the gene responsible for *srn* mutation.

Externally, *srn* mutants exhibit a bent tail as early as 24 hpf, a phenotype that becomes progressively more severe (Fig. 1A), as well as a malformation of the hindbrain, which becomes apparent at 48 hpf (Fig. 1A, brackets). The *srn* locus was mapped between SSLP markers z49730/z14955 and z14614 on chromosome 20, with marker z10756 having no recombinants (Fig. 1B). *Gmds* was found to contain a G to T transversion in the nucleotide sequence that produces a nonconservative glycine (G) to valine (V) substitution of amino acid 178 (G178V) in the short-chain dehydrogenase/reductase (SDR) domain (Fig. 1C, D, E). GMDS is highly conserved at the amino acid level; the fish and human proteins are 87% identical.

*In situ* hybridization showed that from 6 to 12 hpf, *gmds* transcripts are expressed throughout the embryo (Fig. S1A). By 24 hpf, *gmds* transcripts are enriched in the CNS and are also present in somites (Fig. S1B). *Gmds* mRNA expression is present in the CNS at 48 and 72 hpf, with transcripts more abundant in brain than spinal cord (Fig. S1C, D). *Gmds* mRNA is also expressed in the PNS at 72 hpf, including in lateral line neuromasts (data not shown; [27]).

RT-PCR analyses suggested that at least two splice variants exist in zebrafish *gmds*, with or without exon 4, which we name *gmds-L* and *gmds-S* respectively. Both splice variants are expressed in *srn* mutants and WT embryos (Fig. 1F). To confirm that *gmds* is the gene responsible for *srn* phenotypes, both splice variants of the WT and mutant *gmds* cDNAs were fused with *gfp* and were *in vitro* transcribed into mRNA and were injected into 1–2 cell stage embryos collected from *srn* incrosses. In embryos injected with WT *gmds-gfp* mRNAs, 5% were mutant scored by external phenotypes compared to uninjected embryos (29%) or embryos injected with mutant *gmds-gfp* mRNAs (Fig. 1G; one-way ANOVA, followed by Dunn's pairwise comparison, p<0.05). Moreover, when GMDS function was perturbed in WT embryos with a splice-blocking morpholino, all defects seen in *srn* mutants were phenocopied (see below and Fig. S3). These experiments confirm that *gmds* is the gene mutated in *srn*.

### Slytherin mutants exhibit reduced protein fucosylation

**Figure 2:**
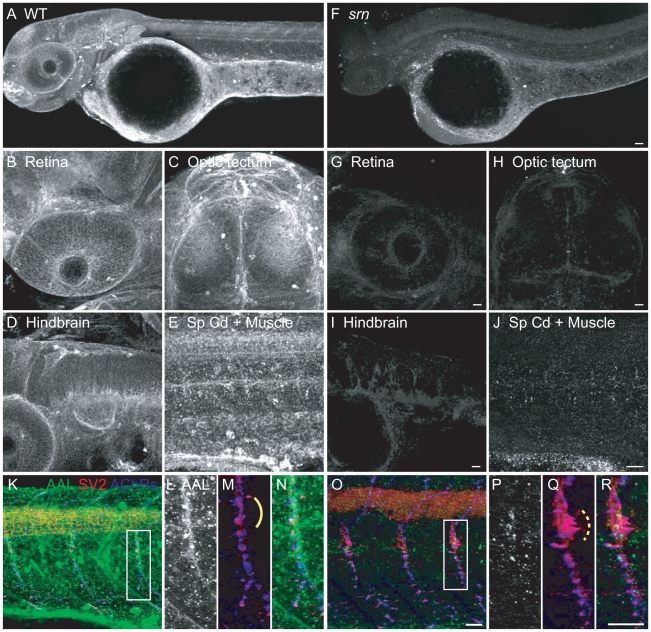
*slytherin* mutants exhibit reduced protein fucosylation as measured by AAL staining. **A.** AAL staining of WT embryos at 48 hpf showed that protein fucosylation is present throughout the embryo (10–15 embryos/2–3 adult pairs for all analyses). **B–E**. Protein fucosylation is prominent in several neural tissues including retina (lateral view), optic tectum (dorsal view), hindbrain (lateral view), spinal cord (lateral view) and neuromuscular synapses (lateral view of axial muscle). **F.** Protein fucosylation is dramatically reduced in *srn* mutants. Scale bar  = 20 µm. **G–J**. Reduced protein fucosylation in several neural tissues. Scale bar  = 20 µm. **K**. Protein fucosylation at neuromuscular synapses in WT embryos at 48 hpf, as shown by the colocalization of AAL staining (green) with markers for presynaptic axons and nerve terminals (SV2, red) and postsynaptic AChR clusters (α-bungarotoxin, blue). **L–N.** Higher magnification of boxed region in **K.** **O.** Protein fucosylation is reduced at *srn* neuromuscular synapses. Scale bar  = 20 µm. **P–R**. Higher magnification of boxed region in **O.** Synapse area is significantly increased in *srn* mutants, e.g., at the choice point (compare dashed bracket in Q to solid bracket in M). Scale bar  = 20 µm.

GMDS is the first enzyme in the *de novo* fucose metabolism pathway, catalyzing the conversion of GDP-D-mannose to GDP-4-keto-6-D-deoxymannose, which is further processed into GDP-fucose [5] and transported into the Golgi where it is used to fucosylate proteins, including Selectins, Notch and many others [5], [28], [29]. Thus AAL staining for fucosylated proteins was performed in 48 hpf WT and mutant embryos (Fig. 2).

In WT embryos, AAL staining was detected in many tissues (Fig. 2A), including olfactory bulb, retina, optic tectum, hindbrain and spinal cord (Fig. 2B–E), which prompted us to examine the potential phenotypes in these structures in *srn*. Moreover, at neuromuscular junctions (NMJ), AAL staining co-localizes with markers for pre- and postsynaptic specializations, such as SV2 and acetylcholine receptors (AChRs) (Fig. 2K–N). In contrast, AAL staining is strongly reduced in *srn* mutants (Fig. 2F–J, O–R), consistent with analyses of cells from CDG IIc patients [3], [4], and of *Drosophila Gfr* mutants [6]. These studies show that protein fucosylation is dramatically reduced in the CNS and other tissues in *srn*, consistent with a loss of function of GMDS, confirming a prediction based on the modeling of the protein crystal structure (see Fig. S2).

### Supplementation with GDP-fucose rescues slytherin phenotypes

**Figure 3:**
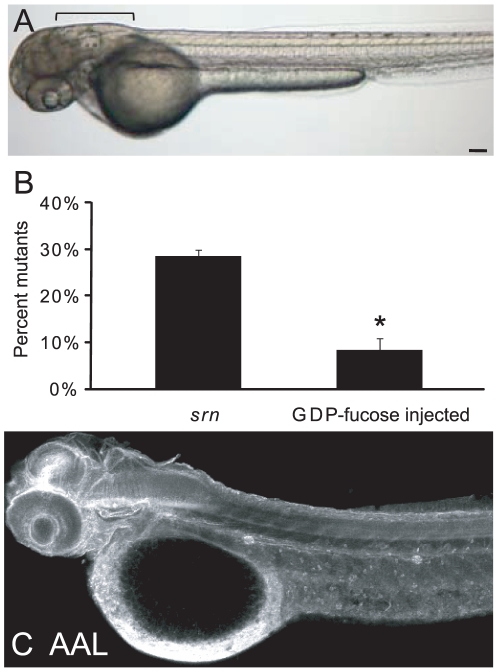
Supplementation with exogenous GDP-fucose rescues *srn* external phenotypes and restores AAL staining. **A.** External *srn* phenotypes including bent tail and aberrant hindbrain (bracket) are rescued by GDP-fucose supplementation (3 embryos). Scale bar  = 100 µm. **B.** GDP-fucose injection significantly reduced the percentage of mutants from 28.6±1.2% to 8.4±2.7% (576 embryos, 4 carrier pairs; Student's test, p<0.0001). **C.** After GDP-fucose supplementation (2 embryos), protein fucosylation as assessed by AAL staining at 48 hpf is rescued throughout *srn* embryos, to levels similar to those in WT embryos. Scale bar  = 100 µm.

Since GMDS functions early in the fucose metabolism pathway, we reasoned that exogenous supply of downstream products may circumvent the genetic defect in *srn*. Therefore, 50 mM GDP-fucose was injected into 1–2 cell stage embryos collected from *srn* incrosses. Compared to uninjected embryos, the percentage of mutant embryos, as scored by external phenotypes (Fig. 3A), was dramatically reduced in GDP-fucose injected embryos (Fig. 3B). Moreover, AAL staining was similar to that in WT embryos at 48 hpf in many if not all tissues (Fig. 3C). Detailed phenotypic analyses further showed that GDP-fucose supplementation is sufficient to rescue neural defects in *srn* mutants (see below and Fig. S3). These strongly suggest that the absence of GDP-fucose, as a result of GMDS dysfunction, is the cause of the *srn* mutant phenotypes, rather than the accumulation of the substrate, GDP-mannose. Thus *srn* mutants display dysregulated protein fucosylation, as is seen in human CDG IIc patients, and that GDP-fucose supplementation restores fucosylation and rescues defects in *srn*.

### Slytherin mutants exhibit defects in neuron and glia number, identity, patterning and axon outgrowth due to Notch-Delta signaling reduction

Our previous work suggested that *srn* exhibited a neurogenic phenotype, specifically an increased number of primary motor neurons [16], similar to that observed in mutants in the Notch-Delta pathway. Analyses of *Drosophila Gfr* mutants suggested that Notch fucosylation is reduced, and that a reduction in Notch signaling might contribute to the pathogenesis in CDG IIc [6]. Therefore, we asked which if any neural defects in *srn* were similar to those observed in mutants in the Notch-Delta pathway or in embryos treated with the γ-secretase inhibitor DAPT to reduce Notch signaling.

We compared *srn* phenotypes with known mutants in the Notch-Delta pathway, *des^b420^* (*deadly seven*, a nonsense mutation in *notch1a* yielding a truncated protein; Gray et al., 2007), *dla^hi781^* (*delta A*, an insertion in *delta A*, predicted to result in a truncated protein [30]) and *mib^hi904^* (*mind bomb*, an insertion in an E3 ligase that targets Delta and other proteins for ubiquitination [13], predicted to result in a truncated protein [31]). Below we describe phenotypes in each mutant in order of increasing disruption of Notch-Delta signaling.

**Figure 4:**
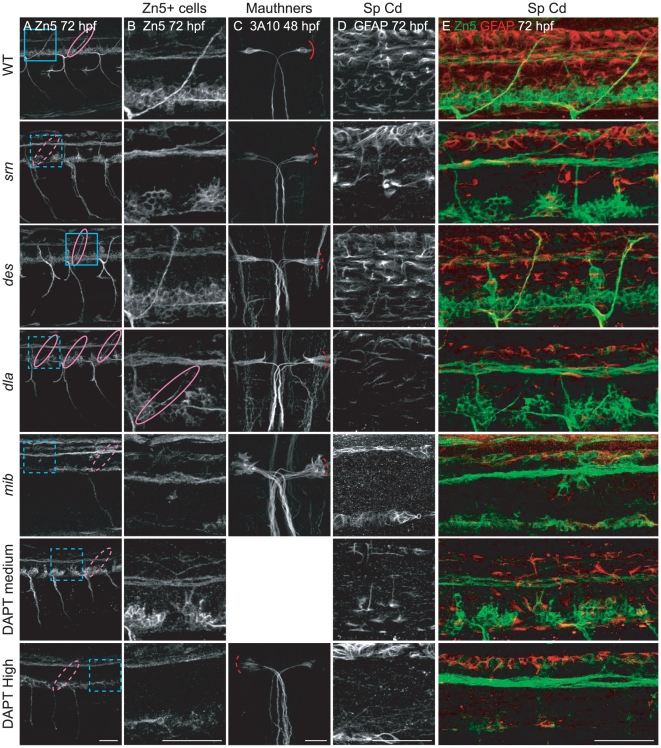
Reduction in Notch-Delta signaling accounts for some *srn* phenotypes. **A**, **B.** Secondary motor neuron cell body number and patterning assayed with Zn5 immunostaining (18 embryos/3 carrier pairs for each). **B.** Higher magnification of boxed region in **A.** At 48–72 hpf, Zn5+ cell number is similar in *srn* and WT (Fig. S4), but the patterning of these cells is aberrant in *srn* embryos. Zn5+ cells are clumped in *srn* mutants (dashed blue bracket) compared to WT embryos (solid blue bracket). *dla* mutants do not have defects in Zn5+ cell number (Fig. S4), but have aberrant Zn5+ cell patterning as in *srn* mutants (dashed blue bracket). *mib* mutants and high dose DAPT treated embryos have aberrant Zn5+ cell number (Fig. S4) and patterning (dashed blue bracket). Medium dose DAPT treated embryos show aberrant Zn5+ cell patterning defects (dashed blue bracket), without an obvious change in cell number (Fig. S4), as in *srn*. The dorsal projecting nerve is absent in *srn* mutants (dashed pink oval) compared to WT (solid pink oval), consistent with increased cell death; this nerve is present in *dla* and *des* mutants (solid pink oval); *des* also has other motor axon pathfinding errors. In *mib* mutants and high and medium dose DAPT treated embryos, the dorsal projecting nerve is absent (dashed pink oval). **C.** In WT embryos at 48 hpf, two Mauthner neurons are present (dorsal view of hindbrain). In *srn*, *des*, *dla*, *mib* and high dose DAPT treated embryos, Mauthner neuron number is increased (dashed red brackets), with the largest increase observed in *mib* (12 embryos, 3 carrier pairs for each). **D.** In the spinal cord, the number of GFAP+ glial cells is reduced in *srn* and *dla* mutants and medium dose DAPT treated embryos compared to WT and *des* embryos at 48–72 hpf. In *mib* and high dose DAPT treated embryos, a more dramatic reduction is observed. The GFAP labeling that remains in *mib* mutants is likely to be in Rohon-Beard neurons dorsally and secondary motor neurons ventrally and is easily separated morphologically and based on its location from glial processes, thus does not interfere with analyses of glial defects (18 embryos, 3 carrier pairs for each). **E.** Overlay showing both the Zn5 and GFAP staining in the spinal cord. Scale bars  = 40 µm.

First, we examined secondary motor neuron cell body number and patterning in the spinal cord, and axon projections in muscle using Zn5 immunostaining. In *srn* mutants at 48 hpf and 72 hpf, while the number of Zn5+ cells is similar between *srn* mutant and WT embryos (Fig. S4), the patterning of these cells is aberrant. Cell bodies are clumped in *srn* mutants (Fig. 4A, B, second panel, dashed blue bracket), compared to evenly spaced cell bodies in WT embryos (Fig. 4A, B, top, solid blue bracket). The dorsally projecting nerve also is absent in *srn* mutants (Fig. 4A, B, second panel, dashed pink oval), consistent with increased Zn5+ cell death [16]. *des* mutants do not have defects in Zn5+ cell number or patterning, but do have motor axon pathfinding errors, possibly due to aberrant formation of somite boundary (Fig. 4A; [9]). *dla* mutants do not have defects in Zn5+ cell number, but have similar aberrant patterning as in *srn* mutants, without the loss of the dorsal projecting nerve (Fig. 4A, B, dashed blue bracket and solid pink oval, respectively). *mib* mutants have aberrant Zn5+ cell number and patterning that is apparent at 48 and 72 hpf, as well as loss of the dorsal nerve.

To analyze the Zn5+ cell patterning defects quantitatively, we counted the number of Zn5+ cells at every 20 µm interval along the rostral-caudal axis of several spinal cord hemisegments. This analysis showed that, while there are 3–5 Zn5+ cells every 20 µm in WT and *des* mutants, there are 1–9 in *srn* and *dla*, and 0–3 in *mib*, confirming our visual impression that patterning is aberrant (Fig. S5E–F). Moreover, while Islet1/2+ cells are dramatically increased in *srn* mutants at 24 hpf, consistent with increased primary motor neurons (Panzer et al., 2005), these cells are decreased at 48 hpf and the majority of Zn5+ cells lack Islet1/2 expression in *srn* mutants (Fig. S5). As Zn5 is expressed in secondary motor neurons and is colocalized with Islet1/2 in wild type embryos, and that Islet1/2 is reduced in Zn5+ cells in *srn*, our results suggest the patterning defects in Zn5+ cells may be correlated with the aberrant Islet1/2 expression. There may be a defect in secondary motor neuron specification in *srn*, consistent with a role for Islet1 and Islet2 in secondary motor neuron formation and axonogenesis [32].

We also found that in the spinal cord, the number of Rohon-Beard neurons is also significantly increased in *srn* mutants at 24 and 48 hpf (Fig. S5A–B), similar to *dla* mutants [33], consistent with reduced Notch-Delta signaling in *srn* mutants.

In the hindbrain and retina, similar defects in neuron number and patterning are present. In the hindbrain at 48 hpf, an increase in Mauthner neurons is observed in *srn*, *des* (as previously reported, Gray et al., 2001), *dla* and *mib*, with the largest increase in Mauthner neuron number observed in *mib* (Fig. 4C, red brackets). Moreover, neuronal patterning in the hindbrain is severely perturbed in *srn* and in *mib* (data not shown). In the retina at 72 hpf, cell number and patterning appear grossly normal in *srn*, *des* and *dla*, but in *mib*, retinal ganglion cell number is reduced (Fig. S6A), probably due to increased cell death, as previously reported [34]. These data suggest that reduced Notch-Delta signaling may account for some of the CNS and PNS phenotypes observed in *srn*.

Because deficiencies in Notch-Delta signaling have been shown to result in reduced gliogenesis [10], [11], [12], [13], we examined glial cells in the spinal cord, hindbrain and retina with GFAP immunostaining. In the spinal cord and hindbrain, the number of GFAP+ glial cells is reduced in *srn* mutants compared to WT embryos at 48–72 hpf (Fig. 4D, 4E and data not shown). A similar reduction in GFAP+ glial cells is also observed in *dla* and *mib*, but not in *des* (Fig. 4D, 4E and data not shown). In the retina, the number of radially oriented GFAP+ Muller cells is decreased in *srn* and *mib*, but not in *des* or *dla* (Fig. S6B). These results suggest that a reduction in Notch-Delta signaling may account for the reduction in glia observed in *srn* mutants.

We then compared *srn* phenotypes with those caused by Notch signaling inhibitor DAPT, a γ-secretase inhibitor, that prevents intramembrane proteolysis of Notch and thus decreases the downstream signaling dependent on the Notch intracellular domain [22]. While high dose of DAPT treatment resulted in phenotypes resembling those seen in *mib* (Fig. 4 and Fig. S6), medium dose DAPT treatment closely recapitulated *srn* phenotypes, including the Zn5+ cell patterning defects and the reduction of GFAP+ glial cells in the spinal cord and retina (Fig. 4; Figs. S5, Fig. S6). These results substantiate the conclusion that a reduction in Notch-Delta signaling may account for the observed neural defects in *srn* mutants.

**Figure 5:**
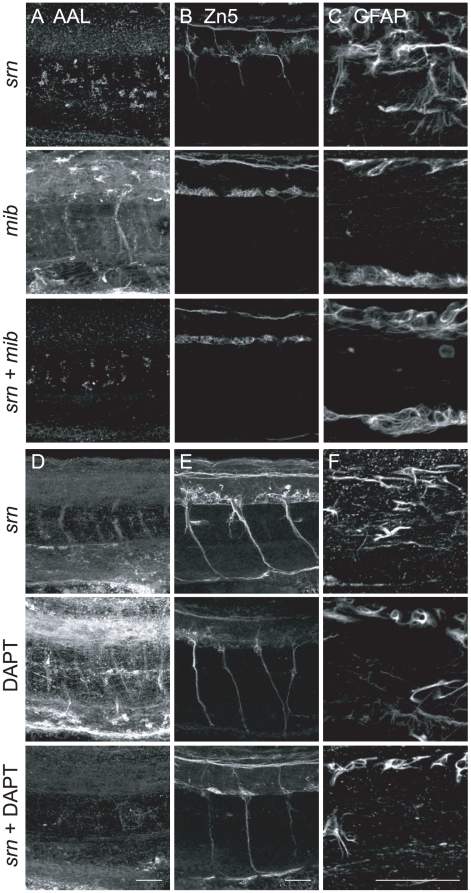
*mib* and DAPT treatment exclude *srn* phenotypes. **A–C.** *mib* excludes *srn* phenotypes. **A.** AAL staining is reduced in *srn* and *srn* + *mib* double mutants, but not in *mib*. **B.** *srn* + *mib* double mutants showed reduction of secondary motor neurons, more severe than *srn* but similar to *mib* alone. **C.** *srn* + *mib* double mutants have reduced GFAP+ glia, more severe than *srn*, but similar to *mib* alone (15 embryos, 2 carrier pairs for each). Scale bar  = 40 µm. **D–F.** DAPT treatment excludes *srn* phenotypes. **D.** AAL staining is reduced in *srn* and *srn* mutants treated with DAPT, but not in DAPT treated embryos. **E.** *srn* mutants treated with DAPT showed reduction of secondary motor neurons, more severe than *srn* but similar to DAPT treated embryos. **F.** *srn* mutants treated with DAPT showed reduction of GFAP+ glia, more severe than *srn*, but similar to DAPT treated WT embryos (10 embryos, 2 carrier pairs for each). Scale bar  = 40 µm.

In order to test the synergy between *srn* and Notch-Delta deficiency, we initially sought to examine embryos double heterozygous for *srn* and *mib*, but these embryos did not show any obvious defects, likely because both single heterozygous embryos are haploid sufficient. We also examined embryos double homozygous for *srn* and *mib*, reasoning since Notch signaling is mostly if not completely absent in *mib* [13], if *srn* defects are also caused by Notch signaling deficiency, introducing *srn* into *mib* background would not result in addictive effects, i.e. would not be more severe then *mib*. Indeed, *srn* and *mib* double mutants showed reduced Zn5+ cells and GFAP+ glial cells in the spinal cord, closely resembling those seen in *mib* (Fig. 5). Furthermore, using the same reasoning, we tested the synergy between *srn* and DAPT treatment. Similarly, in DAPT high dose treated embryos, in which Notch signaling is mostly if not completely blocked, *srn* did not add to the defects caused by DAPT alone, i.e. DAPT treated *srn* mutants resembled DAPT treated WT embryos showing similar reduced Zn5+ cells and GFAP+ glial cells in the spinal cord (Fig. 5). These results are consistent with the hypothesis that Notch signaling deficiency underlies the neurogenesis and gliogenesis defects in *srn*.

**Figure 6:**
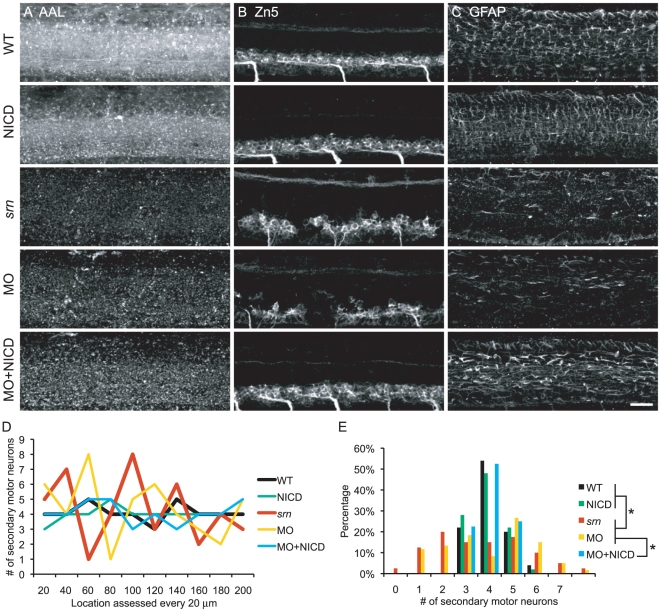
NICD rescues *srn* neuro- and gliogenesis phenotypes. **A.** AAL staining is reduced in *srn*, *gmds* morphants and *gmds* morphants overexpressing NICD, but not in WT embryos or WT embryos overexpressing NICD. **B.** WT and WT overexpressing NICD had normal Zn5+ cell patterning. *srn* and *gmds* MO showed Zn5+ cell patterning defects which was rescued by NICD overexpression in *gmds* morphants. **C.** WT and WT overexpressing NICD had normal GFAP+ glia cells in spinal cord. *srn* and *gmds* morphants had reduced GFAP+ glia cells, rescued by NICD overexpression in *gmds* morphants (>10 embryos in each experiment). Scale bar  = 40 µm. **D–E.** Quantification of Zn5+ cell patterning defects. There are 3–5 Zn5+ cells every 20 µm in WT, WT overexpressing NICD and *gmds* MO overexpressing NICD; compared to 1–8 in *srn* and *gmds* MO embryos. D, data from a representative embryo; E, distribution of all embryos (4–6 embryos; Kolmogorov-Smirnov test, * p<0.05).

If the observed neural defects in *srn* results from reduced Notch signaling, then overexpressing constitutively active Notch would rescue these phenotypes. We utilized transgenic lines in which a constitutively active form of Notch, Notch1a intracellular domain (NICD) is overexpressed under the heat-shock promoter (*Tg(hsp70l:GAL4)*; *Tg(UAS:myc-notch1a-intra)*) [17], recapitulated *srn* phenotypes in these embryos by morpholino knockdown of *gmds* transcripts, and examined whether NICD rescued the neural defects. Indeed, NICD overexpression rescued the Zn5+ cell patterning and reduced GFAP+ glial cells phenotypes in *gmds* morphants (Fig. 6). Moreover, NICD overexpression suppressed the increased mauthner neuron phenotype in *gmds* morphants (Fig. S7). These results strongly suggest that Notch signaling deficiency underlies the neurogenesis and gliogenesis defects in *srn*.

**Figure 7:**
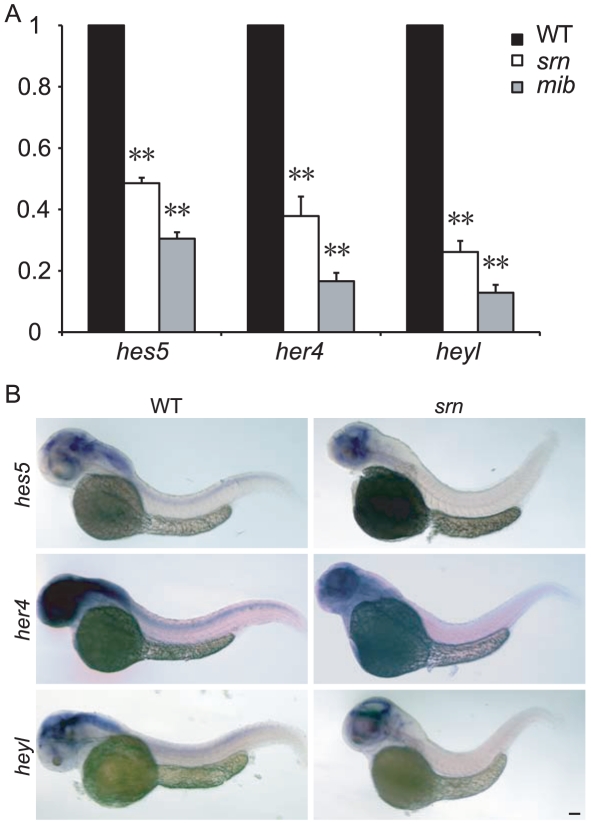
*srn* mutants showed aberrant expression of Notch responsive genes similar to *mib* mutants. **A.** qRT-PCR assessment of fold change in *hes5*, *her4* and *heyl* expression in WT, *srn* and *mib* mutant embryos at 48 hpf, normalized to *β-actin1*. *hes5*, *her4* and *heyl* expression is dramatically reduced in *srn*, similar to those in *mib*, but to a lesser extent. (3–5 experiments, 20 embryos each, one-way ANOVA, Bonferroni's Multiple Comparison Test, ** p<0.001, * p<0.5). **B.** *hes5*, *her4* and *heyl in situ* hybridization at 48 hpf confirm reduced expression in the brain and spinal cord in *srn* mutants compared to WT (>30 embryos for each). Scale bar  = 100 µm.

To further assess whether Notch-Delta signaling is deficient in *srn* mutants, we examined the expression of several Notch effector genes, including *hes5*, *her4* and *heyl* as direct readout of Notch transcriptional activation, using real time quantitative RT-PCR and *in situ* hybridization. *mib* embryos display a strong reduction in Notch signaling [13] and *hes5*, *her4* and *heyl* were collectively shown to be reduced in *mib* mutant fish and/or mice [35], [36], [37], [38], [39]. We found that, at 48 hpf, *hes5*, *her4* and *heyl* expression were significantly reduced in *srn* mutants, similar as in *mib* mutants, although to a lesser extent (Fig. 7A, B). Because these data show that defects in neuron and glia number, patterning and Notch effector genes expression in *srn* mutants are similar to those observed in mutants in the Notch-Delta pathway, a reduction in Notch-Delta signaling caused by the lack of fucosylation accounts for these *srn* phenotypes.

### Slytherin mutants exhibit defects in neuromuscular synaptogenesis due to Notch-Delta signaling reduction

**Figure 8:**
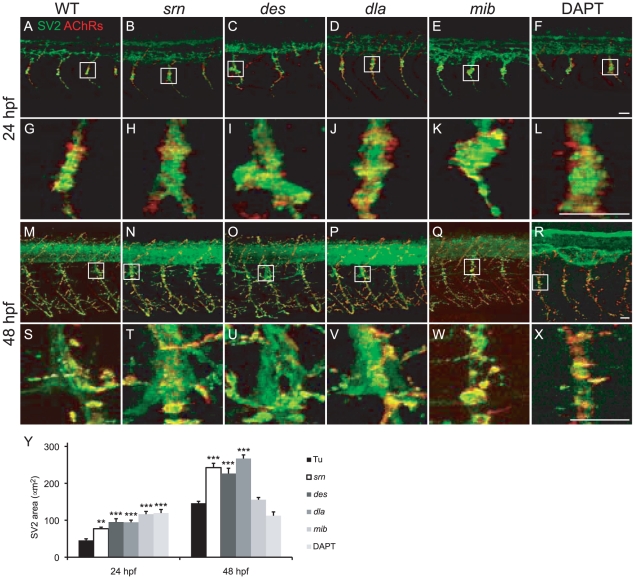
*Slytherin* mutants exhibit defects in neuromuscular synaptogenesis due in part to reduction in Notch-Delta signaling. **A–X.** Presynaptic terminals (green) and postsynaptic AChR clusters (red) in 24 and 48 embryos from WT (A, G, M, S), *srn* (B, H, N, T), *des* (C, I, O, U), *dla* (D, J, P, V), *mib* (E, K, Q, W) and DAPT treated embryos (F, L, R, X). Boxed regions are shown at higher magnification at 24 (G–L) and 48 hpf (S–X; 3 hemisegments in each of 20 embryos, 3 carrier pairs for each). Scale bar  = 20 µm. **Y.** Presynaptic terminal, axon and synapse area at the choice point was significantly increased in all mutants, except in *mib* and DAPT treated embryos at 48 hpf, compared to WT (one-way ANOVA, Bonferroni's Multiple Comparison Test, ** p<0.01, *** p<0.001).

Because *srn* was first identified in a screen for mutants with defects in neuromuscular synaptogenesis, we assessed the role of protein fucosylation and Notch-Delta signaling in neuromuscular synapse formation, particularly at the choice point where the first neuromuscular synapses are made [16]. Choice point neuromuscular synapse size was increased at 24 hpf in *srn*, *des*, *dla*, *mib* and DAPT treated embryos (Fig. 8). At 48 hpf, *mib* and DAPT treated embryos showed no enlargement of choice point neuromuscular synapses, likely due to a reduced number of secondary motor neurons (Fig. S4). These defects are not due to defects in muscle fiber integrity or number (Fig. S8 and [16]). These results show that dysregulated protein fucosylation in *srn* mutants resulted in an aberrant neuromuscular synaptogenesis that was phenocopied in Notch-Delta signaling deficient embryos, suggesting that Notch-Delta signaling plays an important and previously unappreciated role in neuromuscular synapse formation.

### Slytherin mutants exhibit defects in CNS axon branching and synaptic connectivity that are independent of Notch-Delta signaling

**Figure 9:**
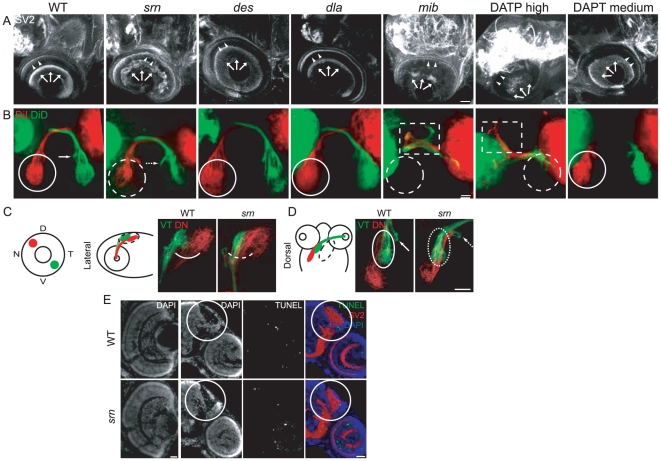
*Slytherin* mutants exhibit defects in axon branching and CNS synaptic connectivity that are independent of Notch-Delta signaling. **A.** In *srn* mutants at 72 hpf, the OPL (arrowheads) and IPL (arrows) are disorganized; this is not seen in *des* or *dla* mutants. In *mib* mutants, retinal ganglion and other cells die, resulting in decreased retina neuropil (rightmost panel; 8 embryos, 2 carrier pairs for each). Scale bar  = 20 µm. **B.** In *srn* mutants, retinal ganglion cell axons grow out to the optic chiasm and to optic tectum, but axon branches are aberrantly distributed within tectum (dashed white circle) and medial axon projections are shifted towards the midline (compare solid arrow and dashed arrow). Virtually all retina was dye labeled, and the labeling pattern was consistent across experiments, thus these defecs aren't due to incomplete dye uptake or labeling in *srn* mutants. These phenotypes are not present in *des* or *dla* mutants, and are also different from *mib* mutants, in which retinal ganglion cell axonal projections to optic tectum are dramatically reduced, as a consequence of retinal ganglion cell death. *Mib* mutants also displayed axon pathfinding errors at the optic chiasm; axons branched anterior to the optic chiasm (dashed square), while branching within tectum was dramatically reduced (dashed white circle; 15 embryos, 3 carrier pairs for each). Scale bar  = 20 µm. **C**, **D.** Topographic mapping of axon projections to optic tectum; dorsonasal (DN) and ventrotemporal (VT) axons were with DiI or DiD. DN and VT axon projections within tectum are aberrant in *srn* mutants, as is overlap dorsally (C) and laterally (D; 8 embryos, 2 carrier pairs for each). Scale bar  = 20 µm. **E.** In retina and optic tectum (white circle), the overall cellular lamination pattern as assessed by DAPI staining is grossly normal in *srn* mutants (compare bottom left panels, WT and *srn*). TUNEL staining showed that increased cell death was observed in the retina of *srn* mutants compared to WT embryos at 72 hpf; no difference in cell death in the optic tectum was observed in *srn* mutants compared to WT embryos at 72 hpf (color overlay, right most panels; 2–3 embryos, 1 carrier pair). Scale bar  = 20 µm.

Phenotypic analyses showed that *srn* has several defects that are not present in mutants in the Notch-Delta pathway *des*, *dla* or *mib*, or DAPT treated embryos. In the retina, while overall cellular lamination is grossly normal in *srn* mutants (Fig. 9E, bottom left panel), neuropil in the outer and inner plexiform layers (OPL and IPL) are dramatically altered (Fig. 9A, arrowheads and arrows). In *srn* mutants at 48–72 hpf, the OPL and IPL synaptic layers are disorganized, and this is not seen in *des*, *dla* or medium dose DAPT treated embryos (Fig. 9A). In *mib* and high dose DAPT treated embryos, retinal ganglion and other cells die, resulting in a reduction in synapses throughout the retina (Fig. 9A). Thus *srn* displayed unique defects in CNS synaptic connectivity that are not phenocopied by Notch signaling deficient embryos. These data are consistent with one of two possibilities. First, fucosylation of proteins other than those involved in Notch-Delta signaling may be required to shape CNS synaptic connectivity. Alternatively, Notch-Delta signaling may contribute, in a specific spatio-temporal context, to these defects. Resolution of these possibilities will require identification of protein targets of *srn*-mediated fucosylation and exploration of their role in CNS synaptic connectivity, and/or analyses of mutants with more precise spatial and temporal disruption of Notch-Delta signaling than are currently available.

Given that AAL staining showed high levels of protein fucosylation in optic tectum (Fig. 2), we examined whether retinal ganglion cell axon outgrowth to and branching within the optic tectum was affected in *srn* and other mutants. In *srn* mutants, retinal ganglion axons grow to the correct location (Fig. 9B), but their axons are aberrantly branched within the optic tectum (Fig. 9B, dashed white circle) and medial axon projections are shifted towards the midline (Fig. 9B, compare solid arrow and dashed arrow). These phenotypes are not present in *des*, *dla* or medium dose DAPT treated embryos (Fig. 9B). In *mib* and high dose DAPT treated embryos, the retinal ganglion cell axon projection to optic tectum is dramatically reduced due to retinal ganglion cell death (Fig. 9B). *Mib* and high dose DAPT treated embryos also displayed retinal ganglion axon pathfinding errors at the optic chiasm (Fig. 9B, dashed square) and decreased branching within the optic tectum (Fig. 9B, dashed white circle). Furthermore, topographic mapping analyses, in which the dorsonasal (DN) and ventrotemporal (VT) retinal ganglion cell projections were differentially labeled (Fig. 9C, D) showed that, in *srn* mutants, the location of the DN and VT axon projections in the optic tectum is aberrant, and that these projections overlap aberrantly dorsally and laterally (Fig. 9D). Moreover, the cellular lamination and cell viability in the optic tectum was similar between *srn* and WT embryos at 72 hpf (Fig. 9E, middle left panels). These results suggest that signaling independent of the Notch-Delta pathway, but requiring protein fucosylation, modulates axon branching and synaptic patterning in the CNS.

## Discussion

We report that the *srn* mutation causes a loss of GMDS function, leading to a severe reduction in protein fucosylation, including that of Notch among many others. *Srn* displays increased neurogenesis, decreased gliogenesis, increased neuronal cell death, abnormal neuronal patterning, abnormal axon arborization, and abnormal neuromuscular and CNS synaptic connectivity, indicating that protein fucosylation plays an important role in several aspects of neural development.

### Notch-Delta signaling reduction underlies some but not all srn neural phenotypes

Our results suggest that both Notch-dependent and -independent mechanisms contribute to the neural phenotypes observed in *srn*. *Srn* mutants showed reduced Notch transcriptional activity, as assayed by *hes5*, *her4* and *heyl* expression, increased primary motor neuron, Rohon-Beard neuron and Mauthner neuron number, decreased gliogenesis and abnormal neural patterning. These defects are phenocopied by mutants in the Notch-Delta pathway and in embryos with reduced Notch signaling. That *mib* and Notch signaling inhibition by DAPT occlude *srn* defects, and that NICD overexpression rescues these *srn* phenotypes, strongly suggest that the dysregulated fucosylation of proteins in the Notch-Delta pathway accounts for these prominent neural defects in *srn* mutants. While the lack of anti-zebrafish Notch antibodies prevented direct analysis of Notch fucosylation, Notch is known to be fucosylated, and other proteins in the Notch-Delta pathway, including Delta, Serrate and Jagged, contain consensus sequence(s) for O-linked fucose modification [40], [41], [42]. Notch is also N-fucosylated, in which fucose is added to N-linked glycan side chains [6], [43], [44]. Notch O- and N-fucosylation has been shown to be reduced in the Drosophila *Gfr* null [6]. It thus seems highly likely that the fucosylation of proteins in the Notch-Delta pathway is aberrant in *srn* mutants and that this accounts for some, but not all, *srn* neural phenotypes.

Interestingly, there is a hierarchy in the spectrum of phenotypes among *srn* and mutant in the Notch-Delta pathway. Phenotypes in *des*, except for the axon pathfinding errors, are weaker than those in *dla*, and both of these are weaker than *srn*. This is consistent with the hypothesis that many Notch-Delta factors, including Notch, Delta, Serrate and Jagged, require proper protein fucosylation and compromised fucosylation of these proteins may account for the wider spectrum of defects seen in *srn*. *Mib* mutants also displayed a wide range of defects, not seen in the other three mutants, both due to the fact that *mib* regulates a large spectrum of Notch signaling, as it interacts with various Notch ligands and is broadly required for Notch signaling in many tissues, and also it interacts with a number of proteins besides Delta and may serve as an integrator of multiple neuronal developmental pathways [45].

Moreover, our observation that *srn* and mutants in the Notch-Delta pathway have increased neuromuscular synapses supports a previously underappreciated role for Notch-Delta signaling during synaptogenesis. Because primary motor neuron number is increased in *srn*, it is difficult to separate direct effects of Notch-Delta signaling on presynaptic differentiation from indirect effects on neurogenesis. The total number of motor neurons innervating trunk muscles actually decreases due to secondary motor neuron cell death [16], while the increase in neuromuscular synapse number and size persists. This strongly suggests that Notch-Delta signaling plays a role in synaptogenesis, independent of its role in neurogenesis.

Recent work has shown that reduced protein fucosylation, as a result of *gmds* mutation in *twohead* (*twd*) mutants, results in defects in the migration of vagus motor neuron progenitors [46]. However, they argued that Notch signaling is unaltered, based on several lines of evidence. First, they concluded from semi-quantitative RT-PCR analyses, that expression of *her4*, a downstream effector in the Notch pathway, was unchanged, but their data suggests that *her4* expression may indeed be decreased. On the other hand, we show using quantitative RT-PCR in our Fig. 7 that *her4* is reduced in *srn* mutants. Second, Ohata et al. analyzed motor neuron number and patterning by *in situ* for *islet1* and *islet2* and concluded that motor neuron number and patterning are unaltered in *twd*. On the other hand, we show that the number of neurons assayed by *islet1* and *islet2 in situ* [16] and by islet1/2 immunostaining at 24 hpf (Fig. S5) is increased in *srn* mutants. Thus, detailed analyses of neural and glial phenotypes and analyses of additional Notch target genes in *twd* mutants may help resolve this apparent discrepancy.

Previous work suggested that Fringe, a glycosyltransferase that glycosylates specific sites on the Notch extracellular domain during its intracellular processing, modulates Notch activity [29], [47], [48], [49]. In *srn*, both O- and N-fucosylation are compromised due to reduced production of fucose moieties. Fringe acts one step downstream of O-fucosylation, adding N-glycans onto fucosylated sites. We speculate that Fringe loss of function may result in similar, but milder, deficits than in *srn* mutants. Indeed, recent work suggested that *lunatic fringe* (*Lfng*), a known modifier of Notch, promotes the lateral inhibition of neurogenesis, that *Lfng* loss of function by morpholino knockdown leads to increased expression of proneural genes and increased neurogenesis, and that transgenic overexpression of *Lfng* decreases neurogenesis [50]. These observations are consistent with our results, and further support our conclusion that dysregulated glycosylation of Notch and its ligands results in Notch signaling deficiency and leads to increased neurogenesis.

While deficiencies in Notch-Delta signaling underlie some *srn* phenotypes, other *srn* phenotypes are likely to be independent of this pathway. *Srn* mutants exhibit prominent defects in retinotectal connectivity that are quite different from those observed in mutants in the Notch-Delta pathway such as *des* and *dla* in which no defects in retinotectal axon branching are observed, and from the dramatic reduction in retinal ganglion cell number and axon pathfinding observed in *mib*. We present several lines of evidence that support the conclusion that some, but clearly not all, of the mechanisms underlying the neural phenotypes in *srn* are Notch-dependent. Future work will focus on identifying the fucosylated proteins that mediate the neural deficits that are independent of Notch-Delta signaling.

It seems likely that the regulation of Notch signaling by fucosylation is context dependent, i.e. different aspects of neural development require specific types and extent of fucosylation and other modifications of Notch receptors and/or ligands, in a particular spatiotemporal fashion. Our results do not completely rule out the possibility that Notch signaling may contribute, in a specific spatiotemporal context, to the synaptic defects and retinal ganglion cell arborization defects in *srn*, and this will only be resolved once the relevant fucosylation targets are identified.

### Srn as a zebrafish model for congenital disorders of glycosylation

Over the last decade, a large number of human genetic diseases with aberrant glycoprotein synthesis have been identified and grouped as congenital disorders of glycosylation (CDG). Since glycosylation is essential for the function of many proteins, it is not surprising that disruption of glycosylation can lead to severe, multi-systemic phenotypes, including neurodevelopmental and cognitive disorders. In *srn* mutants, the *gmds* mutation largely abolishes the synthesis of GDP-fucose, resulting in reduction or elimination of both O-linked and N-linked fucosylation of Notch and many other proteins. Thus it is possible that disruption of O- as well as N-linked glycosylation of Notch and other proteins contributes to CDG IIc pathogenesis, although this has not been examined extensively in humans.

There are several reports of neural deficits in CDGIIc patients, including severe mental retardation, microcephaly, cortical atrophy, seizures, psychomotor retardation and hypotonia [2], [4], [51]. These clinical observations are consistent with the CNS and PNS cellular phenotypes observed in *srn*. Giving the advantage of performing imaging, genetic and pharmacological manipulations in zebrafish, *srn* will be a useful tool to guide future analyses in human CDG IIc patients and contribute to a better understanding of the mechanisms responsible for this devastating disorder that affects nervous system and other organ development.

## Supporting Information

Figure S1 Gmds mRNA localization by in situ hybridization in wild type zebrafish embryos from 12 to 72 hpf. In situ hybridization was performed as described previously (Panzer et al., 2005), with anti-sense (A–E) gmds probe; sense probe was used as a control (F). Several hundred embryos from several carrier pairs were used from 6 to 72 hpf. A. From 6 to 12 hpf, gmds transcripts are expressed throughout the embryo. B. By 24 hpf, gmds transcripts are highly expressed in the CNS and are also expressed in somites at lower levels. C, D. Gmds mRNA expression is present in the CNS at 48 (C) and 72 (D) hpf, with transcripts more abundant in brain than spinal cord.
(2.49 MB EPS)

Figure S2 Modeling of zebrafish GMDS protein structure. Because of the high degree of amino acid sequence conservation between zebrafish and human GMDS, we reasoned that it would be informative to superimpose the zebrafish GMDS sequence onto the human GMDS crystal structure; this was done using a search of the using a search of the Protein Data Bank database (www.pdb.org) and MODELLER and PYMOL software. The wild type (brown rods) and srn (blue rods) primary amino acid sequence was modeled onto the human GMDS protein crystal structure. A: As in the srn mutation, Valine was substituted for Glycine at residue 178 and an energy minimization calculation was performed. When the srn mutation is present, the Valine deforms a nearby Glutamate residue, Glu155. This change is predicted to push away the substrate GDP-manose, resulting in loss of function. B: To understand how the movement of Glu155 could affect surrounding amino acids, the wild type structure (brown sticks) was examined in more detail. Three ordered H2O molecules exist between the negatively charged group on Glu155 and the negatively charged phosphate group on GDP. The bond lengths between water oxygens and phosphate or carboxylic acid oxygens are appropriate to form hydrogen bonds to coordinate GDP to Glu155.
(0.83 MB EPS)

Figure S3 GDP-fucose rescue of srn and morpholino knockdown of gmds. A. RT-PCR showed >80% of gmds transcript was mis-spliced after gmds morpholino (4 ng) injection. B–E. External phenotypes in srn and gmds morphants (E) include tail bend (compare B, wild type with C, srn) which is rescued after GDP-fucose supplementation (D). F–I. srn (G) and gmds morphants (I) showed reduced AAL staining compared to wild type (F) which is rescued after GDP-fucose supplementation (H). J–M. srn (K) and gmds morphants (M) showed increased Mauthner neuron number compared to wild type (J) a phenotype that is rescued after GDP-fucose supplementation (L). N–Q. srn (O) and gmds morphants (Q) showed reduced GFAP+ glia in the spinal cord compared to wild type (N), a phenotype that is rescued after GDP-fucose supplementation (P). R–U. srn (S) and gmds morphants (U) showed increased neuromuscular synapses compared to wild type (R), a phenotype that is rescued after GDP-fucose supplementation (T). Scale bar  = 40 µm. In each experiment, at least 10 srn, normal siblings, gmds morphants or GDP-fucose rescued srn mutant embryos were assessed at 48 hpf. These results show that GDP-fucose rescues external and neural defects in srn mutants and that gmds knockdown by morpholino phenocopies srn phenotypes. Together, these further support the conclusions that gmds is the gene mutated in srn, that the fucose metabolism pathway is deficient in srn mutants, and that the resulting lack GDP-fucose is the cause of the srn mutant phenotypes, rather than the accumulation of the substrate, GDP-mannose.
(6.55 MB EPS)

Figure S4 Zn5+ cell number is reduced in mib but not srn, des or dla compared to wild type embryos. The number of Zn5+ cells was counted from embryos at 48 and 72 hpf after immunostaining with Zn5 antibody and confocal reconstruction of the motor neuron pool. At 48 hpf, Zn5+ cell number per hemisegment was similar among wild type (46±2), srn (49±1), des (52±2) and dla (46±2) embryos, and is significantly reduced in mib mutant embryos (27±2) (1 hemisegment in each of 6–9 48 hpf embryos counted of each genotype; one-way ANOVA, Bonferroni's Multiple Comparison Test, only mib is significantly different compared to other mutants and wild type, p<0.001). At 72 hpf, Zn5+ cell number per hemisegment was similar among wild type (63±2), srn (62±1), des (63±2) and dla (61±2) embryos, and is significantly reduced in mib mutant embryos (36±2) (1 hemisegment in each of 6–10 72 hpf embryos counted of each genotype; one-way ANOVA, Bonferroni's Multiple Comparison Test; only mib is significantly different compared to other mutants and wild type, * p<0.001).
(0.37 MB EPS)

Figure S5 Immunostaining of Zn5, Islet1/2 and GFAP, and quantification of Zn5+ cell patterning defects. A. At 24 hpf, Islet1/2 staining is increased in srn mutants compared to WT embryos, in primary motor neurons and Rohon-Beard neurons, identified based on their morphology and location in the spinal cord. Dashed lines indicate segment boundaries. B. At 48 hpf, in WT embryos, Zn5+ cells are also Islet1/2+. In srn mutants, Islet1/2 expression is reduced and majority of Zn5+ cells are not Islet1/2+. C. Zn5 and GFAP immunostaining in WT and srn mutants at 48 hpf, showing the spatial relationship between these markers. D. GFAP staining in HuC:GFP embryos at 48 hpf, showing the spatial relationship between neuron cell bodies and GFAP+ processes in the spinal cord. Scale bar  = 40 µm. E–H. Quantification of Zn5+ cell patterning defects. There are 3–5 Zn5+ cell at every 20 µm interval in WT and des mutants, 1–9 in srn, dla mutants and medium dose DAPT treated embryos, and 0–3 in mib and high dose DAPT treated embryos, consistent with clumping and gaps in the spinal cord. Data from a representative embryos is shown in E and G. The distribution of all embryos is shown in F and H (4–9 embryos for each; Kolmogorov-Smirnov test, p<0.05).
(5.80 MB EPS)

Figure S6 Reduction in Notch-Delta signaling accounts for some srn phenotypes in the retina. A. Retina patterning was examined with immunostaining using antibody Zn5 at 72 hpf. Retina cell patterning appears grossly normal in srn, des, dla and medium dose DAPT treated embryos, but in mib and high does DAPT treated embryos retinal ganglion cell number is reduced, probably due to increased cell death, as previously reported (Bernardos et al., 2005) (8 embryos, 2 carrier pairs were examined). Scale bar  = 40 µm. B. Glial cells in the retina were examined after immunostaining with anti-GFAP antibody. In the retina, the number of radially oriented GFAP+ Muller cells is decreased in srn and mib and medium dose DAPT treated embryos, but not in des or dla (8 embryos, 2 carrier pairs were examined). Scale bar  = 40 µm. These results suggest that a reduction in Notch-Delta signaling may account for the glial defects observed in srn mutants.
(2.16 MB EPS)

Figure S7 NICD overexpression suppresses the increased Mauthner neuron phenotype in gmds morphants. A. AAL staining is reduced in gmds morphants overexpressing NICD, but not in WT embryos or WT embryos overexpressing NICD. B. WT embryos have a pair of Mauthner neurons. WT overexpressing NICD showed dramatic hindbrain patterning defects, resulting in an almost complete loss of Mauthner neurons. gmds morphants overexpressing NICD showed similar reduction of Mauthner neurons. This result suggests NICD overexpression suppresses the increase of Mauthner neurons observed in gmds morphants and thus supports our conclusion that reduction of Notch-Delta signaling in srn mutants is responsible for the neurogenesis defects (3–4 embryos assessed for each manipulation). Scale bar  = 20 µm.
(2.95 MB EPS)

Figure S8 Muscle patterning is grossly normal in srn mutants. Slow muscle fibers were examined with F59 antibody and glia cells in the spinal cord were examined with GFAP antibody. While there is an obvious reduction of GFAP+ glia cells in the spinal cord in srn mutants, the patterning of slow muscle fibers is similar in srn and wild type embryos at 48 hpf. Previous work showed that fast muscle fiber number and patterning are unaltered in srn compared to wild type embryos at 48 hpf (Panzer et al., 2005; 3 embryos, 1 carrier pair were examined). Scale bar  = 200 µm.
(1.86 MB EPS)
